# Resilience as a Predictor of Satisfaction and Well-Being in Nursing Clinical Education: A Cross-Sectional Study

**DOI:** 10.3390/nursrep16040120

**Published:** 2026-04-02

**Authors:** Denise Rodriguez Medrano, Viola Cisari, Emanuela Morenghi, Daniela Cattani, Simone Cosmai, Giovanni Cangelosi, Sara Morales Palomares, Mauro Parozzi, Stefano Mancin, Fabio Petrelli, Diego Lopane, Beatrice Mazzoleni

**Affiliations:** 1 Department of Biomedical Sciences, Humanitas University, Via Rita Levi Montalcini 4, 20072 Milan, Italy; deniserodriguez0506@icloud.com (D.R.M.); viola.cisari03@gmail.com (V.C.); beatrice.mazzoleni@hunimed.eu (B.M.); 2 IRCCS Humanitas Research Hospital, Via Alessandro Manzoni 56, 20089 Milan, Italy; emanuela.morenghi@humanitas.it (E.M.); daniela.cattani@humanitas.it (D.C.); simone.cosmai@gavazzeni.it (S.C.); stefano.mancin@humanitas.it (S.M.); diego.lopane@hunimed.eu (D.L.); 3 School of Pharmacy, Experimental Medicine and “Stefania Scuri” Public Health Department, University of Camerino, Via Madonna delle Carceri 9, 62032 Camerino, Italy; petrelli@unicam.it; 4 Department of Pharmacy, Health and Nutritional Sciences, University of Calabria, Via Pietro Bucci, 87036 Rende, Italy; sara.morales@unical.it; 5 Department of Medicine and Surgery, University of Parma, Via Volturno 39, 43125 Parma, Italy

**Keywords:** resilience, nursing students, psychological well-being, clinical education, cross-sectional study, Italy

## Abstract

**Background/Aims**: Resilience is a protective factor that helps nursing students manage the challenges of clinical education. However, the relationships between resilience, clinical internship satisfaction, and psychological well-being remain underexplored. To examine the associations between resilience, satisfaction with clinical internships, and psychological well-being among undergraduate nursing students across academic years and campuses. **Methods**: A cross-sectional observational study was conducted. A total of 302 undergraduate nursing students from four campuses of a northern Italian university completed three validated instruments: the 14-item Resilience Scale (RS-14), the Clinical Learning Quality Evaluation Index (CLEQI), and the 12-item General Health Questionnaire (GHQ-12). Descriptive, correlational, and multiple regression analyses were performed. **Results**: Resilience was positively associated with clinical learning satisfaction and inversely associated with psychological distress. Regression models confirmed resilience as a significant predictor of both clinical satisfaction (*p* < 0.01) and psychological well-being (*p* < 0.05), adjusting for age and gender. **Conclusions**: Resilience plays a crucial role in improving both educational satisfaction and psychological outcomes in nursing students. Integrating resilience-building strategies into nursing curricula could enhance learning experiences and well-being.

## 1. Introduction

Resilience has been defined as the ability to maintain or regain a positive level of functioning in the face of stressors, representing a dynamic and proactive process that enables individuals to effectively adapt to adverse circumstances [1]. In academic settings, this construct assumes a developmental and evolving dimension, shaped by the interaction between individual characteristics and the educational environment [2]. Nursing education, internationally recognized as one of the most demanding university programs, exposes students to substantial emotional, cognitive, and physical demands, particularly during clinical placements [3]. For many students, the clinical setting represents the first direct contact with illness, suffering, and death, requiring not only technical skills but also considerable emotional stability and adaptive capacity [4].

The emotional burden of caregiving, the fear of making mistakes, integration into unfamiliar teams, and the management of complex procedures all contribute to the risk of psychological distress, anxiety, and burnout [5,6]. These stressors are further compounded by global challenges—such as economic instability and geopolitical tensions—that amplify the uncertainty experienced by university students [7,8]. Combined with academic pressures, these factors can compromise mental well-being and educational performance, as highlighted in studies conducted in European and Mediterranean contexts [9].

Within this scenario, resilience emerges as a key protective resource for supporting mental health and academic success [10]. It enables students not only to withstand stress but also to transform educational experiences into opportunities for growth, particularly when the learning environment is well-structured and supportive [11]. However, when the clinical environment is perceived as hostile or inconsistent, resilience alone may not suffice to mitigate distress, potentially fueling a vicious cycle of stress and demotivation [12,13].

Satisfaction with clinical internships is a multi-determined outcome influenced by supervision quality, learning opportunities, perceived safety and care standards, self-directed learning, and the overall clinical environment [14].

Among the factors influencing the quality of clinical education, the clinical mentor plays a pivotal role [15]. Effective mentorship is associated with increased confidence, reduced anxiety, and improved clinical competence in nursing students [16]. Conversely, poor supervision quality and lack of mentor availability are primary contributors to clinical dissatisfaction and early attrition from nursing programs [17].

Despite increasing attention to post-pandemic student well-being, Italian research on nursing students’ resilience remains limited and fragmented, with few studies integrating psychological and clinical variables in a comprehensive analysis [18]. Some studies have focused on perceived stress or on the quality of the clinical learning environment [4,14], but an integrated perspective exploring resilience, mental health, and clinical placement satisfaction simultaneously is still lacking. Psychological well-being, in the context of nursing education, refers to a multi-dimensional construct encompassing emotional functioning, absence of distress symptoms, and capacity to maintain adaptive functioning under academic and clinical demands. Satisfaction with clinical placements reflects students’ subjective appraisal of the quality of their learning environment, encompassing supervision quality, learning opportunities, safety standards, self-directed learning, and clinical environment [14].

### Aims

In light of this background, the present study aims to investigate resilience levels among undergraduate nursing students and to explore their relationship with general psychological well-being and clinical placement satisfaction. Understanding these associations may help in designing tailored educational strategies, promoting academic retention, and supporting the development of professional identity. The primary objective of the study is to explore perceived resilience levels among first-, second-, and third-year nursing students. The secondary objective is to examine the correlation between resilience, psychological well-being, and satisfaction with clinical training. The research would answer to the main question: “What is the relationship between resilience, psychological well-being, and clinical placement satisfaction among nursing students?”.

## 2. Materials and Methods

### 2.1. Study Design

A cross-sectional observational design was adopted, conducted between June and July 2024 across four campuses of a university located in northern Italy, each situated in different geographical regions of the country. The study was structured in accordance with the STROBE reporting guidelines [19] and the STROBE checklist was completed/Supplementary Table S1). Data collection was carried out through an anonymous online questionnaire administered via Google Forms during debriefing sessions following clinical placements. Participants received a direct link via institutional email. The digital format ensured secure and rapid access, encouraged voluntary participation, and safeguarded personal data in compliance with GDPR (EU Regulation 2016/679).

### 2.2. Sample

The study was conducted across four campuses of the same university in Italy, all offering a Bachelor of Science in Nursing program. All students enrolled in the first, second, or third year during the 2023/2024 academic year who had completed their required clinical placements were eligible to participate. A consecutive, non-probability sampling method was adopted.

### 2.3. Study Variables

The variables collected were categorized into three main areas. The first included sociodemographic data—such as age and gender—and academic information regarding the year of study, campus location, type of high school diploma obtained, and any history of repeating academic years. The second area focused on perceived resilience, considered the primary outcome variable of the study. Lastly, measures of general psychological well-being and satisfaction with the clinical placement experience were collected and treated as explanatory or predictive variables in the analysis model.

### 2.4. Data Collection Instruments

#### 2.4.1. Resilience Scale

Perceived resilience was assessed using the Resilience Scale (RS-14) in Italian validation. This 14-item tool uses a 7-point Likert scale (from 1 = “strongly disagree” to 7 = “strongly agree”) to measure individuals’ ability to cope with and overcome adverse situations. It is widely used in educational and youth contexts to evaluate overall psychological resilience. Total scores range from 14 to 98, with standardized cut-offs used to classify resilience into five categories: very low, low, moderate, moderately high, and high [20].

#### 2.4.2. 12-Item General Health Questionnaire

General psychological well-being was measured using the 12-item General Health Questionnaire (GHQ-12) in its Italian validation [21]. This self-administered tool evaluates psychological distress symptoms experienced in the past weeks, covering emotional, relational, and functional aspects. Total GHQ-12 scores range from 0 to 12 under the bimodal method. Participants are classified as: normal well-being (0–2), mild distress (3–5), moderate distress (6–8), and severe distress (9–12) [22].

#### 2.4.3. Clinical Learning Quality Evaluation Index

Satisfaction with clinical placements was assessed using the Clinical Learning Quality Evaluation Index (CLEQI), a tool specifically developed and validated for nursing students in Italy [14]. The CLEQI comprises 25 items distributed across five subscales (5 items each): Supervision (SUP), Learning Opportunities (LO), Safety and Quality of Care (SQC), Self-Directed Learning (SDL), and Clinical Environment (CE). Each item is rated on a 4-point Likert scale (0 = never; 3 = always). Total score ranges from 0 to 75, with higher scores indicating greater satisfaction; subscale scores range from 0 to 15. Internal consistency in the present sample was excellent (Cronbach’s α = 0.93), consistent with the original validation (α = 0.94). Permission for use was obtained from the development team prior to data collection [14].

### 2.5. Inclusion and Exclusion Criteria

Eligible participants included all students enrolled in the first, second, or third year of the Bachelor of Science in Nursing program during the 2023/2024 academic year who had completed their scheduled clinical placements. Inclusion criteria were: (a) active participation in the academic program, (b) completion of the placement at one of the participating campuses, and (c) sufficient proficiency in the Italian language to understand and complete the questionnaire.

Exclusion criteria included: (a) prolonged absence from clinical activities during the academic year, (b) failure to provide digital informed consent, and (c) incomplete questionnaire submission. A consecutive, non-probabilistic sampling strategy was used to recruit participants.

### 2.6. Strategies to Manage Risk of Bias

All questionnaires were administered digitally via a secure online platform to ensure participant anonymity and to facilitate data collection across geographically distributed campuses [14].

To minimize the risk of bias, several methodological precautions were implemented. The tools were administered in a standardized, anonymous digital format, without the involvement of teaching staff in data collection, to avoid perceived pressure or influence. Questionnaires were completed at the end of the clinical training period, during debriefing sessions, allowing for free reflection on the experience and reducing the impact of hierarchical relationships. No incentives were offered for participation. These measures ensured procedural neutrality and improved the reliability of the collected data.

### 2.7. Statistical Analysis

To verify the adequacy of the sample size relative to the observed effects, a post hoc power analysis was conducted using G*Power 3.1 software [23]. Results indicated that for a medium effect size (r = 0.30; f^2^ = 0.15), a sample of 302 participants provides a statistical power exceeding 99%, with a type I error rate set at α = 0.05 (Appendix A). Descriptive statistics were used to summarize sociodemographic and academic characteristics of the sample, including means, standard deviations, and absolute and relative frequencies. The distribution of continuous variables was tested for normality to ensure the appropriateness of parametric tests. A one-way analysis of variance (ANOVA) was used to compare mean resilience scores across years of study and university campuses, followed by Tukey’s post hoc tests to detect significant group differences. Pearson correlation coefficients were calculated to explore linear relationships between all tool adopted, both in the overall sample and in stratified subgroups. To evaluate the predictive capacity of psychological well-being and clinical satisfaction on resilience levels, multiple linear regression models were constructed, controlling for age and gender. These models were also applied separately to specific subgroups (year of study and campus) to identify any differential patterns in the associations. The analysis also addressed a potential paradox observed between resilience and psychological distress, with particular attention to inverse correlations detected in certain subgroups. This aimed to further investigate the adaptive role of resilience in high-pressure clinical settings, even in the presence of significant subjective distress. All statistical analyses were conducted using Stata 18 software [24].

### 2.8. Ethical Considerations

The study was approved by the Ethics Committee of the participating university (Protocol code: CLI_RIC_12). All participants provided digital informed consent before accessing the questionnaire, which included clear and comprehensible information about the study objectives, participation procedures, and data management. Anonymity and voluntary participation were guaranteed, with no coercion or incentives provided. The entire research protocol adhered to the ethical principles outlined in the Declaration of Helsinki, in its latest version approved by the World Medical Association in 2013 [25], ensuring responsible and ethically sound data collection, handling, and storage practices.

## 3. Results

**Figure 1:**
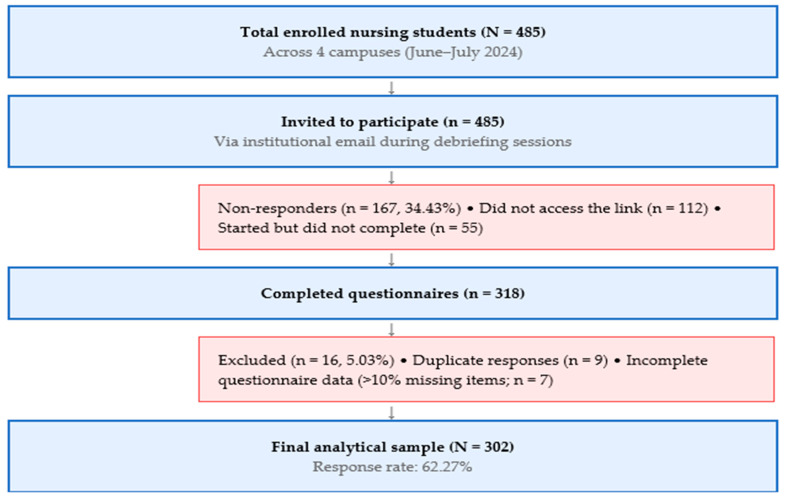
Participant selection flowchart. Note. Duplicate responses were identified through Google Workspace single-response settings and cross-checked via IP address matching. Questionnaires with >10% missing items across the three instruments (RS-14, GHQ-12, CLEQI) were excluded from analysis, consistent with recommended thresholds for scale validation studies. Legend N = total population; n = sample size at each stage; % = percentage of initial invited population.

A total of 302 undergraduate nursing students completed the questionnaire, yielding a response rate of 62.27%. Of the 485 nursing students invited across four university campuses, 318 completed the online questionnaire. After excluding 16 responses due to duplication (*n* = 9) or incomplete questionnaire data (>10% missing items across RS-14, GHQ-12, or CLEQI; *n* = 7), the final analytical sample comprised 302 participants (response rate: 62.27%; Figure 1). The sample was predominantly female (74.83%, *n* = 226) and consisted mainly of students aged 21–25 years (56.95%, *n* = 172). Participants were distributed across Rozzano-Milan (42.38%), Bergamo (22.19%), Castellanza (19.21%), and Catania (16.23%), and represented all three academic years (Table 1). Most students held only a high school diploma (94.70%), and 14.24% reported having repeated at least one academic year.

### 3.1. Resilience Levels

RS-14 scores showed overall moderate levels of perceived resilience. RS-14 scores showed a non-linear trajectory across academic years. A notable decrease was observed from Year 1 to Year 2, followed by a significant increase in Year 3, suggesting that the second year of clinical training may represent a particularly challenging transition period for students’ perceived resilience. Mean scores ranged from 67.5 (second year, Bergamo) to 80.4 (third year, Castellanza). First-year students in Catania also reported high values (79.6 ± 13.3), among the highest in the entire sample (Table 2).

### 3.2. Psychological Well-Being

Psychological well-being, measured through the GHQ-12, indicated a widespread presence of psychological distress. Mean scores indicated the presence of mild to moderate distress categories across most participants, with a tendency toward higher distress levels in the more advanced academic years. Participants were classified using the validated bimodal categories: normal well-being (0–2), mild distress (3–5), moderate distress (6–8), and severe distress (9–12). The highest mean scores were observed in the second and third years at the Castellanza campus (19.1 ± 4.6 and 19.0 ± 4.3, respectively), indicating greater psychological strain in this setting (Table 3).

Overall, psychological well-being tended to decline with academic progression, contrasting with the increasing levels of resilience (Supplementary Figure S1).

### 3.3. Clinical Internship Satisfaction

Satisfaction with clinical placements, assessed through the CLEQI, was generally positive. Total mean scores ranged from 36.8 ± 13.9 (second year, Castellanza) to 52.7 ± 12.7 (first year, Catania). Overall, first-year students reported higher levels of satisfaction compared to upper-year students, with a noticeable decline in the second year across several campuses (Table 4). The most appreciated dimensions were learning opportunities and the clinical environment, while the dimension related to safety and quality of care received comparatively lower scores (Supplementary Figure S2).

### 3.4. Correlations Between Resilience, Clinical Satisfaction and Psychological Well-Being

Statistical analysis revealed significant associations between resilience and the explanatory variables explored. A positive correlation between resilience and CLEQI emerged in specific subgroups. Among first-year students at Rozzano-Milan, higher CLEQI scores were associated with higher resilience levels (β = 0.453, *p* = 0.001). Similar results were observed among second-year students at Castellanza (β = 0.396, *p* = 0.039) and Bergamo (β = 1.003, *p* = 0.016).

Additionally, age emerged as a significant predictor of resilience among first-year students at Rozzano-Milan (β = 1.580, *p* = 0.015), whereas gender showed no significant effect (Supplementary Figure S4).

Notably, some analyses revealed significant inverse correlations between resilience and psychological well-being. Specifically, among first- and third-year students in Bergamo, and first-year students in Rozzano-Milan, higher resilience scores were associated with increased GHQ-12 scores—indicating greater psychological distress (r = −0.577, *p* = 0.004; r = −0.635, *p* = 0.001; r = −0.304, *p* = 0.038).

These findings suggest that in certain contexts, elevated resilience levels may coexist with a heightened subjective perception of psychological distress (Supplementary Figures S4–S6). While resilience may support students in adapting and persisting through demanding academic and clinical settings, it does not necessarily alleviate the psychological burden they experience. This paradox warrants further investigation, particularly to clarify the underlying mechanisms linking resilience and mental health outcomes in nursing education.

## 4. Discussion

Our findings reveal a nuanced relationship between resilience development and psychological well-being among nursing students during clinical training. While resilience scores progressively increased across academic years—consistent with developmental models of adaptive capacity [26,27]—this growth did not uniformly translate into improved psychological well-being. In specific cohorts, higher resilience coexisted with elevated distress levels, suggesting that resilience may function as a survival mechanism under sustained stress rather than an indicator of flourishing [28,29]. This paradox challenges traditional assumptions linking resilience to positive mental health outcomes and underscores the context-dependent nature of adaptive coping in high-demand educational environments. Geographic and institutional variability emerged as significant moderators of resilience trajectories. First-year students in Catania demonstrated unexpectedly high baseline resilience, potentially reflecting organizational culture, peer support structures, or regional attitudes toward healthcare education [30,31]. Conversely, mid-program fluctuations observed in Bergamo and Castellanza may indicate critical transition periods where academic demands intensify without proportional support scaling—a phenomenon documented in longitudinal studies of healthcare student wellbeing [32]. These patterns highlight the role of contextual factors in shaping adaptive responses and suggest that institutional climate may buffer or amplify stressors inherent to clinical training. The positive association between resilience and clinical placement satisfaction—particularly in dimensions of learning opportunities and clinical environment quality—aligns with emerging evidence that supportive educational contexts activate protective mechanisms [33]. Students reporting high-quality supervision, accessible learning resources, and psychologically safe clinical spaces exhibited both greater resilience and lower distress, suggesting that institutional investments in clinical learning infrastructure yield dual benefits for competence development and mental health [34,35]. This finding reinforces calls for embedding resilience-enhancing strategies within nursing curricula, including reflective practice, simulation-based learning in protected environments, and structured debriefing following emotionally intensive clinical encounters [36,37].

The inverse relationship between resilience and psychological well-being observed in certain subgroups warrants particular attention. The persistence of psychological distress alongside increasing resilience scores invites re-examination of what resilience measures in this population. Drawing on the distinction between ‘recovery resilience’ (return to pre-stress baseline) and ‘resistance resilience’ (maintenance of functioning under ongoing stress) [38], our findings are more consistent with the latter. Students may develop the capacity to sustain academic and clinical performance while the subjective experience of distress remains unresolved—a ‘functional but not flourishing’ state [39] that can mask underlying exhaustion and risk progression toward burnout. Drawing on stress-coping frameworks [32], we interpret this pattern as reflecting “compensatory resilience”—wherein individuals develop adaptive strategies in response to chronic adversity without experiencing subjective relief from distress. This mechanism may be especially pronounced in healthcare education, where professional socialization pressures encourage perseverance despite emotional burden [27]. Recent studies in nursing student populations corroborate this phenomenon, demonstrating that resilience can coexist with burnout when systemic stressors remain unaddressed [40,41,42,43,44,45]. Our findings thus challenge simplistic resilience interventions and call for parallel investments in structural supports—reduced clinical workload, psychological counselling access, and organizational policies addressing workplace stressors. The observed decrease in resilience scores between Year 1 and Year 2 is consistent with ‘transition shock’ models [46], describing a period of heightened vulnerability as students confront the gap between idealized expectations and clinical realities. The second year often marks intensification of clinical hours, increased responsibility, and reduced peer support structures, without proportional increases in coping resources.

Satisfaction with clinical internships showed an overall positive but variable pattern. The Supervision and Learning Opportunities subscales received the highest ratings, while Safety and Quality of Care received comparatively lower scores. The notable CLEQI decline in the second year—particularly at Castellanza—parallels the resilience trajectory and may reflect compounding effects of a more demanding clinical environment without adequate supervisory support

The four campuses differ in several potentially relevant organizational features. The Rozzano-Milan campus is embedded within a large IRCCS tertiary referral center with high clinical case complexity. The Bergamo campus operates within a mid-size teaching hospital with a strong tradition of simulation-based nursing education. Castellanza is located within a private hospital group with distinct organizational culture. Catania represents a geographically and socio-culturally distinct context. While we do not have validated data on student-to-mentor ratios or formal organizational culture assessments for this study, these structural differences likely contribute to the observed campus-level variability and represent a priority for future comparative research.

### 4.1. Implications for Nursing Education

At the curricular level, curricula should incorporate: (1) mindfulness-based stress reduction (MBSR) protocols, which have demonstrated efficacy in reducing anxiety and improving emotional regulation in pre-registration students; (2) high-fidelity simulation debriefings explicitly focused on emotional processing, using the Debriefing for Meaningful Learning (DML) framework; (3) structured peer-support programs, including dyadic mentorship pairing senior and junior students, which have been shown to buffer transition stress and (4) reflective writing exercises integrated into clinical log requirements, supporting metacognitive processing of emotionally intensive clinical experiences [41].

Such interventions should be developmentally sequenced, with foundational emotional regulation skills introduced early and advanced coping strategies layered as clinical complexity increases [33,40]. At the institutional level, universities must prioritize clinical learning quality through enhanced supervision models, transparent communication channels between academic and clinical partners, and systematic monitoring of placement conditions [14]. The observed association between clinical environment quality and student resilience suggests that improving placement infrastructure—including preceptor training, student-to-supervisor ratios, and access to psychological support—may yield measurable benefits for both learning outcomes and mental health [11,12]. Policy implications extend to accreditation standards and regulatory frameworks governing nursing education. Mandating routine assessment of student wellbeing using validated instruments (e.g., GHQ-12, burnout inventories) could enable early identification of at-risk cohorts and trigger institutional responses [36]. Similarly, establishing benchmarks for clinical placement quality—informed by tools such as the CLEQI—may incentivize healthcare organizations to invest in student-supportive environments [14] (Summary Table 5).

### 4.2. Perspectives for Future Research and Practice

Advancing understanding of resilience in nursing education requires methodological and conceptual innovation. Longitudinal designs tracking students from enrollment through early professional practice would clarify whether resilience gains observed during training translate into career sustainability and job satisfaction [27]. Qualitative approaches—including narrative interviews and phenomenological analysis—could illuminate the lived experiences underlying quantitative patterns, particularly the mechanisms through which students develop resilience despite persistent distress [41]. Future investigations should explore the intersection of resilience with burnout, compassion fatigue, and professional identity formation [36]. Given evidence that healthcare students experience burnout rates comparable to practicing clinicians [42], interventions targeting resilience must be evaluated for their capacity to prevent—not merely cope with—chronic occupational stress. Experimental studies comparing resilience curricula (e.g., mindfulness-based stress reduction, cognitive-behavioral approaches, peer mentorship models) would inform evidence-based program development [33]. From a practice perspective, healthcare organizations hosting nursing students should recognize their role as educational partners—not merely clinical sites. Structured onboarding programs, dedicated student support staff, and transparent feedback mechanisms may enhance both learning quality and student wellbeing [11,14]. Establishing communities of practice linking academic faculty, clinical preceptors, and students could facilitate knowledge exchange and collaborative problem-solving around clinical training challenges.

### 4.3. Strengths and Limitations

This study’s strengths include its multi-campus design, adequate sample size (N = 302), and use of validated instruments (RS-14, GHQ-12, CLEQI), which enhance generalizability and methodological rigor. The integration of resilience, psychological wellbeing, and clinical placement satisfaction within a single framework addresses a gap in European nursing education research. However, several limitations warrant acknowledgment. The cross-sectional design precludes causal inference and limits understanding of resilience development over time. While our sample represents four Italian university campuses, findings may not generalize to public universities, international contexts, or non-traditional student populations. The reliance on self-report measures introduces potential response bias, and the absence of objective performance indicators (e.g., clinical competency assessments, academic outcomes) limits our ability to evaluate resilience’s functional impact. Additionally, our study did not assess potentially confounding variables such as prior mental health history, social support networks, financial stressors, or personality traits (e.g., neuroticism, conscientiousness) known to influence resilience and wellbeing. The observed resilience-distress paradox may reflect unmeasured third variables or complex mediation pathways requiring structural equation modeling or path analysis. Finally, the study’s timing—conducted during post-pandemic recovery—may have influenced findings. Students who enrolled during or immediately after COVID-19 restrictions may exhibit distinct resilience profiles compared to pre-pandemic cohorts, limiting historical comparability.

Given the cross-sectional design, causal directionality cannot be established. While our regression models position resilience as a predictor of both clinical satisfaction and psychological well-being, the inverse pathway is equally plausible: high-quality clinical environments characterized by effective supervision and psychological safety may foster and reinforce students’ resilience capacity. Future longitudinal and experimental designs are needed to disentangle these reciprocal relationships.

## 5. Conclusions

This study examined resilience as a predictor of clinical internship satisfaction and psychological well-being among nursing students at four campuses of a northern Italian university. Resilience was positively associated with clinical learning satisfaction in several subgroups and inversely associated with psychological distress, confirming its role as a protective construct. However, the non-linear trajectory and its paradoxical coexistence with elevated distress challenge simple additive models of resilience as a uniformly protective factor. Resilience among nursing students develops progressively but does not guarantee psychological wellbeing—particularly when institutional supports fail to match clinical demands. High-quality learning environments, characterized by effective supervision and psychological safety, emerge as critical protective factors. Nursing education must move beyond individual resilience training toward systemic reforms addressing workload, mental health access, and clinical placement quality. Longitudinal research integrating resilience, burnout, and professional identity is essential to building sustainable healthcare workforces equipped for modern practice complexities.

## Figures and Tables

**Table 1:** Sample characteristics (*n* = 302).

Variable	Category	*n* (%)
Gender	Female	226 (74.83%)
	Male	71 (23.51%)
	Not specified	5 (1.66%)
Age Group	18–20 years	69 (22.85%)
	21–25 years	172 (56.95%)
	26–30 years	30 (9.93%)
	>30 years	28 (9.27%)
Academic Background	First degree	286 (94.70%)
	Previous degree	16 (5.30%)
Repeated Academic Year	Yes	43 (14.24%)

Legend. *n* = total sample size; *n* = number of participants in each category; % = percentage of total sample.

**Table 2:** Scale RS-14 results by campus and year.

Campus	Year	*n*	Mean ± SD	Very Low	Low	Moderate	Moderately High	High
Milan	1st	47	71.0 ± 13.9	7	6	15	7	2
	2nd	50	68.1 ± 15.5	15	2	10	15	1
	3rd	42	73.1 ± 11.2	5	3	12	12	2
Bergamo	1st	23	72.4 ± 9.4	1	2	11	6	0
	2nd	22	67.5 ± 20.3	4	1	8	4	1
	3rd	25	73.1 ± 12.1	2	3	5	8	0
Castellanza	1st	26	70.1 ± 11.2	2	7	9	3	1
	2nd	27	67.8 ± 15.6	3	6	9	5	1
	3rd	17	80.4 ± 9.9	0	1	3	4	4
Catania	1st	23	79.6 ± 13.3	1	3	4	6	7

Note and Legend. Categories reflect standardized RS-14 cut-offs: very low (≤56), low (57–64), moderate (65–73), moderately high (74–81), and high (≥82) levels of resilience. *n* = number of participants; SD = standard deviation; Mod. High = moderately high resilience level.

**Table 3:** GHQ-12 results by campus and year.

Campus	Year	*n*	Mean ± SD	Normal	Moderate Distress	Severe Distress
Milan	1st	47	16.8 ± 4.9	17	15	15
	2nd	50	17.3 ± 4.2	12	24	14
	3rd	42	17.9 ± 4.3	8	24	10
Bergamo	1st	23	17.2 ± 4.1	6	9	8
	2nd	22	17.1 ± 5.5	8	7	7
	3rd	25	15.9 ± 6.6	11	7	7
Castellanza	1st	26	17.7 ± 3.5	3	16	7
	2nd	27	19.1 ± 4.6	3	13	11
	3rd	17	19.0 ± 4.3	1	9	7
Catania	1st	23	16.1 ± 5.4	8	9	6

Psychological well-being is categorized using the bimodal scoring method (range 0–12) as: normal well-being (0–2), mild distress (3–5), moderate distress (6–8), and severe distress (9–12).

**Table 4:** CLEQI by campus and academic year.

Campus	Year	*n*	Total Score (Mean ± SD)	SUP	LO	SQC	SDL	CE
Milan	1st	47	47.8 ± 13.3	2.20 ± 0.73	2.21 ± 0.76	2.26 ± 0.65	1.74 ± 0.78	2.35 ± 0.68
	2nd	50	42.7 ± 16.2	2.02 ± 0.82	2.02 ± 0.78	2.04 ± 0.72	1.63 ± 0.88	1.82 ± 0.92
	3rd	42	47.5 ± 14.7	2.26 ± 0.69	2.36 ± 0.74	2.03 ± 0.57	1.87 ± 0.72	2.01 ± 0.92
Bergamo	1st	23	45.6 ± 13.2	1.99 ± 0.69	2.22 ± 0.65	2.17 ± 0.54	1.59 ± 0.84	2.30 ± 0.82
	2nd	22	40.4 ± 12.5	1.83 ± 0.62	1.88 ± 0.66	2.01 ± 0.73	1.58 ± 0.63	1.80 ± 0.78
	3rd	25	47.5 ± 10.2	2.17 ± 0.62	2.34 ± 0.60	2.15 ± 0.53	1.87 ± 0.56	2.07 ± 0.80
Castellanza	1st	26	44.9 ± 16.4	2.02 ± 0.84	2.10 ± 0.76	2.31 ± 0.65	1.64 ± 0.81	2.00 ± 0.94
	2nd	27	36.8 ± 13.9	1.69 ± 0.74	1.78 ± 0.73	1.79 ± 0.62	1.32 ± 0.75	1.60 ± 0.84
	3rd	17	51.9 ± 12.3	2.35 ± 0.66	2.51 ± 0.63	2.31 ± 0.53	2.04 ± 0.73	2.47 ± 0.68
Catania	1st	23	52.7 ± 12.7	2.32 ± 0.66	2.57 ± 0.50	2.49 ± 0.86	2.04 ± 0.85	2.43 ± 0.69

Note and Legend. CLEQI total scores and sub-dimension scores among nursing students at four university campuses across three academic years. All scores are reported as mean ± standard deviation. Sub-dimensions: SUP = Supervision (quality of clinical supervision received); LO = Learning Opportunities (availability and quality of learning experiences); SQC = Safety and Quality of Care (perceived safety standards and care quality in clinical settings); SDL = Self-Directed Learning (opportunities for autonomous learning and initiative); CE = Clinical Environment (overall quality of the clinical learning environment). Sub-dimension scores range from 0 to 3, with higher scores indicating greater satisfaction. *n* = number of participants; SD = standard deviation; CLEQI = Clinical Learning Quality Evaluation Index.

**Table 5:** Summary of educational and institutional implications.

Level	Proposed Action	Evidence Base
Curricular	MBSR protocols; DML debriefing; reflective writing	Dreifuerst, 2012 [47]; Lopane et al., 2025 [41]
Institutional	Enhanced supervision models; preceptor training; student-to-supervisor ratios	Palese et al., 2019 [14]; Jokelainen et al., 2011 [16]
Policy	Routine GHQ-12/burnout monitoring; CLEQI-based placement benchmarks	Merino-Godoy et al., 2022 [36]; present study

Note. MBSR = Mindfulness-Based Stress Reduction; DML = Debriefing for Meaningful Learning; GHQ-12 = 12-item General Health Questionnaire; CLEQI = Clinical Learning Quality Evaluation Index.

## Data Availability

The data presented in this study are available on reasonable request from the corresponding authors. The data are not publicly available due to ethical and privacy restrictions, as they contain information that could compromise the confidentiality of the participants.

## Supplementary Materials

The following supporting information can be downloaded at: https://www.mdpi.com/article/10.3390/nursrep16040120/s1, Table S1: STROBE Statement—Checklist of items for cross-sectional observational studies; Figure S1: Scatter plot of resilience versus psychological well-being stratified by academic year (Rozzano-Milan campus); Figure S2: Scatter plot of resilience versus psychological well-being stratified by academic year (Bergamo campus); Figure S3: Scatter plot of resilience versus psychological well-being stratified by academic year (Castellanza campus); Figure S4: Scatter plot of resilience versus clinical learning quality (CLEQI) stratified by academic year (Rozzano-Milan campus); Figure S5: Scatter plot of resilience versus clinical learning quality (CLEQI) stratified by academic year (Bergamo campus); Figure S6: Scatter plot of resilience versus clinical learning quality (CLEQI) stratified by academic year (Castellanza campus).

## Public Involvement Statement

No public involvement in any aspect of this research.

## Guidelines and Standards Statement

This manuscript was drafted in accordance with the Strengthening the Reporting of Observational Studies in Epidemiology (STROBE) guidelines for cross-sectional observational studies (von Elm et al., 2007) [19].

## Appendix A

### Appendix A.1. Measurement Instruments—Italian-Language Questionnaire Battery

The following questionnaires were administered in Italian to all participants, in accordance with the validated Italian versions of each instrument. Translations of item wording into English are provided for reference purposes only. All statistical analyses were performed on the original Italian responses. Permissions for use of all three instruments were obtained from the respective copyright holders prior to data collection.

**Table A1:** Overview of the three measurement instruments used in the study.

Instrument	Abbrev.	Items	Response Scale	Reference
Resilience Scale	RS-14	14	7-point Likert (1–7)	Wagnild, 2009 [48]; Italian validation: Callegari et al., 2016 [20]
General Health Questionnaire	GHQ-12	12	4-point (bimodal scoring 0-0-1-1)	Goldberg & Williams, 1988 [49]; Italian validation: Piccinelli & Politi P, 1993 [21]
Clinical Learning Quality Evaluation Index	CLEQI	22	4-point Likert (0–3)	Palese et al., 2019 [14]

Note: Abbrev. = abbreviation. Internal consistency (Cronbach’s α) for each instrument in the present study sample: RS-14 α = 0.92; GHQ-12 α = 0.85; CLEQI α = 0.93.

### Appendix A.2. Resilience Scale (RS-14)—Italian Version

Original authors: Wagnild GM (2009) [48]. A review of the Resilience Scale. *Journal of Nursing Measurement*, 17(2), 105–113. https://doi.org/10.1891/1061-3749.17.2.105.
Italian validation: Callegari C. et al. (2016) [20]. Reliability and validity of the Italian version of the 14-item Resilience Scale. Psychol Res Behav Manag. 2016 Oct 3;9:277–284. doi: 10.2147/PRBM.S115657.
Psychometric properties in this sample: Cronbach’s α = 0.92. Scoring: sum of all 14 items (range 14–98). Higher scores indicate greater perceived resilience. Cut-off categories: Very Low ≤ 56; Low 57–64; Moderate 65–73; Moderately High 74–81; Moderately High-High 82–90; High > 90.

Instructions (Italian, as administered): “Indica il tuo grado di accordo con ciascuna delle seguenti affermazioni, scegliendo un valore da 1 (Fortemente in Disaccordo) a 7 (Fortemente d’Accordo)”.
Instructions (English translation): “Indicate your level of agreement with each of the following statements, choosing a value from 1 (Strongly Disagree) to 7 (Strongly Agree)”.

**Table A2:** RS-14 item wording in Italian (as administered) and English translation.

#	Italian Wording (as Administered)	English Translation (for Reference Only)
1	Di solito riesco a cavarmela in un modo o nell’altro	When I make plans, I follow through with them.
2	Mi sento orgoglioso/a per le cose che ho realizzato nella mia vita	I usually manage one way or another.
3	Di solito faccio le cose senza il minimo sforzo, seguendo i miei tempi	I am able to depend on myself more than anyone else.
4	Sono amico/a di me stesso/a	Keeping interested in things is important to me.
5	Sento di poter gestire molte cose allo stesso tempo	I can be on my own if I have to.
6	Sono determinato/a	I feel proud that I have accomplished things in my life.
7	Posso affrontare momenti difficili perché ne ho già fatto esperienza in precedenza	I usually take things in stride.
8	Ho auto-disciplina	I am friends with myself.
9	Mantengo interesse nelle cose	I feel that I can handle many things at a time.
10	Di solito trovo qualcosa per cui sorridere	I am determined.
11	Il credere in me stesso/a mi aiuta a superare i momenti difficili	I seldom wonder what the point of it all is.
12	In una situazione di emergenza c’è qualcuno su cui posso contare	I take things one day at a time.
13	La mia vita è piena di significato	I can get through difficult times because I’ve experienced difficulty before.
14	Quando mi trovo in situazioni difficili di solito trovo da solo/a un modo per uscirne	I have self-discipline.

Note: Response scale: 7-point Likert (1 = Strongly Disagree to 7 = Strongly Agree). English translations are provided for international readers; all data collection and scoring used the Italian version.

### Appendix A.3. General Health Questionnaire (GHQ-12)—Italian Version

Original authors: Goldberg DP & Williams P (1988) [49]. *A User’s Guide to the General Health Questionnaire.* Windsor: NFER-Nelson.
Italian validation: Piccinelli, M. (1993) [21]. Struttura fattoriale della versione a 12 domande del General Health Questionnaire in un campione di giovani maschi adulti. *Epidemiologia e Psichiatria Sociale*. 1993;2(3):173–181. doi:10.1017/S1121189X00006990.
Scoring method: Bimodal method (0-0-1-1): response options 1 and 2 = 0; options 3 and 4 = 1. Total score range: 0–12. Classification: Normal well-being (0–2); Mild distress (3–5); Moderate distress (6–8); Severe distress (9–12) [50]. Psychometric properties in this sample: Cronbach’s α = 0.85.

Instructions (Italian, as administered): “Le seguenti domande riguardano la sua salute in generale nelle ultime settimane. Per favore, risponda a tutte le domande semplicemente segnando la risposta che, a suo giudizio, meglio descrive la sua situazione attuale. Si ricordi che vuole conoscere i problemi presenti o recenti, non quelli che ha avuto in passato”.

**Table A3:** GHQ-12 item wording in Italian (as administered), English translation, and response options.

#	Italian Wording (as Administered)	English Translation	Response Options (Italian/English)
1	Durante le ultime settimane, ti sei sentito/a: In grado di concentrarsi su ciò che stava facendo?	During the past few weeks, have you felt: Been able to concentrate on what you are doing?	1. Più del solito/Better than usual 2. Come al solito/Same as usual 3. Meno del solito/Less than usual 4. Molto meno del solito/Much less than usual
2	Durante le ultime settimane, ti sei sentito/a: Di aver perso molto sonno tanto da preoccuparsi?	During the past few weeks, have you felt: Lost much sleep over worry?	1. Per niente/Not at all 2. Non più del solito/No more than usual 3. Piuttosto più del solito/Rather more than usual 4. Molto più del solito/Much more than usual
3	Durante le ultime settimane, ti sei sentito/a: Di essere produttivo (aver fatto tante cose) nella maggior parte delle attività?	During the past few weeks, have you felt: Felt that you are playing a useful part in things?	1. Più del solito/Better than usual 2. Come al solito/Same as usual 3. Meno del solito/Less than usual 4. Molto meno del solito/Much less than usual
4	Durante le ultime settimane, ti sei sentito/a: In grado di prendere decisioni nella maggior parte dei casi?	During the past few weeks, have you felt: Felt capable of making decisions about things?	1. Più del solito/Better than usual 2. Come al solito/Same as usual 3. Meno del solito/Less than usual 4. Molto meno del solito/Much less than usual
5	Durante le ultime settimane, ti sei sentito/a: Costantemente sotto pressione?	During the past few weeks, have you felt: Felt constantly under strain?	1. Per niente/Not at all 2. Non più del solito/No more than usual 3. Piuttosto più del solito/Rather more than usual 4. Molto più del solito/Much more than usual
6	Durante le ultime settimane, ti sei sentito/a: Di non essere in grado di superare le difficoltà?	During the past few weeks, have you felt: Felt that you couldn’t overcome your difficulties?	1. Per niente/Not at all 2. Non più del solito/No more than usual 3. Piuttosto più del solito/Rather more than usual 4. Molto più del solito/Much more than usual
7	Durante le ultime settimane, ti sei sentito/a: In grado di ritagliarsi del tempo libero e goderne?	During the past few weeks, have you felt: Been able to enjoy your normal day-to-day activities?	1. Più del solito/Better than usual 2. Come al solito/Same as usual 3. Meno del solito/Less than usual 4. Molto meno del solito/Much less than usual
8	Durante le ultime settimane, ti sei sentito/a: In grado di risolvere i suoi problemi?	During the past few weeks, have you felt: Been able to face up to your problems?	1. Più del solito/Better than usual 2. Come al solito/Same as usual 3. Meno del solito/Less than usual 4. Molto meno del solito/Much less than usual
9	Durante le ultime settimane, ti sei sentito/a: Infelice o depresso/a?	During the past few weeks, have you felt: Been feeling unhappy and depressed?	1. Per niente/Not at all 2. Non più del solito/No more than usual 3. Piuttosto più del solito/Rather more than usual 4. Molto più del solito/Much more than usual
10	Durante le ultime settimane, ti sei sentito/a: Come se avesse perso la fiducia in se stesso/a?	During the past few weeks, have you felt: Been losing confidence in yourself?	1. Per niente/Not at all 2. Non più del solito/No more than usual 3. Piuttosto più del solito/Rather more than usual 4. Molto più del solito/Much more than usual
11	Durante le ultime settimane, ti sei sentito/a: Come se avesse minore stima di sé?	During the past few weeks, have you felt: Been thinking of yourself as a worthless person?	1. Per niente/Not at all 2. Non più del solito/No more than usual 3. Piuttosto più del solito/Rather more than usual 4. Molto più del solito/Much more than usual
12	Durante le ultime settimane, ti sei sentito/a: Con uno stato emotivo nel complesso felice?	During the past few weeks, have you felt: Been feeling reasonably happy, all things considered?	1. Più del solito/Better than usual 2. Come al solito/Same as usual 3. Meno del solito/Less than usual 4. Molto meno del solito/Much less than usual

Note: Bimodal scoring (0-0-1-1): options 1–2 = 0; options 3–4 = 1. Total range: 0–12. Items 2, 5, 6, 9, 10, 11 use negatively-worded response options; items 1, 3, 4, 7, 8, 12 use positively-worded options. Higher scores indicate greater psychological distress.

### Appendix A.4. Clinical Learning Quality Evaluation Index (CLEQI)—Italian Version

Authors: Palese, A. et al. (2019) [14]. Clinical Learning Quality Evaluation Index per la valutazione della qualità dell’apprendimento clinico degli studenti infermieri e raccomandazioni di utilizzo. Medicina e Chirurgia. 83, 3685–3693, 2019. DOI: 10.4487/medchir2019-83-3.
Structure: 22 items organized across five factors (see below). Response scale: 4-point Likert (0 = Not at all/Per nulla; 1 = Somewhat/Abbastanza; 2 = Very much/Molto; 3 = Extremely/Moltissimo). Total score range: 0–66 (higher scores = greater satisfaction with clinical learning). Psychometric properties in this sample: Cronbach’s α = 0.93. Permission for use was obtained from the development team prior to data collection.

Instructions (Italian, as administered): “Il questionario esplora la qualità dell’esperienza di tirocinio clinico. Per ciascuna affermazione, indichi quanto spesso ha sperimentato quanto descritto durante l’ultimo tirocinio.” (0 = Per nulla; 1 = Abbastanza; 2 = Molto; 3 = Moltissimo).

**Table A4:** CLEQI item wording in Italian (as administered) and English translation, by factor.

Factor	Item	Italian Wording (as Administered)	English Translation (for Reference Only)
Factor 1—Quality of Clinical Supervisory Strategies (Items 1–6)			
1	1	Il tutor ha esplicitato i ragionamenti che sottendevano le decisioni assistenziali	The clinical supervisor explained the reasoning underlying care decisions.
1	2	Il tutor mi poneva domande che mi aiutavano nel ragionamento clinico	The clinical supervisor asked me questions that helped my clinical reasoning.
1	3	Ho avuto la possibilità di condividere con il tutor le emozioni provate durante l’esperienza di tirocinio	I had the opportunity to share with the supervisor the emotions experienced during my clinical internship.
1	4	Il tutor ha mediato la mia relazione con i pazienti/famigliari quando la situazione era difficile	The clinical supervisor mediated my relationship with patients/families when the situation was difficult.
1	5	Il tutor era entusiasta di insegnarmi la pratica infermieristica	The clinical supervisor was enthusiastic about teaching me nursing practice.
1	6	Nella valutazione finale, il tutor è stato/a coerente con i feedback che mi ha fornito durante il tirocinio	In the final evaluation, the clinical supervisor was consistent with the feedback provided during the internship.
Factor 2—Learning Opportunities (Items 7–12)			
2	7	Ho percepito fiducia nei miei confronti	I felt trusted.
2	8	Ho potuto sperimentarmi in autonomia nelle attività	I was able to practise activities autonomously.
2	9	Mi è stato affidato un adeguato livello di responsabilità	I was assigned an appropriate level of responsibility.
2	10	Ho avuto la possibilità di esprimere le mie opinioni e riflessioni critiche	I had the opportunity to express my opinions and critical reflections.
2	11	Mi sono sentito/a rispettato/a come studente	I was respected as a student.
2	12	Sono stato/a incoraggiato/a nei momenti di difficoltà	I was encouraged during difficult moments.
Factor 3—Safety and Quality of Care (Items 13–16)			
3	13	Gli infermieri avevano buoni standard di pratica professionale	Nurses demonstrated good standards of professional practice.
3	14	Era garantita la sicurezza dei pazienti/residenti/ospiti	Patient/resident/guest safety was guaranteed.
3	15	I dispositivi di protezione individuali e di sicurezza erano accessibili	Individual protective devices and safety equipment were accessible.
3	16	Gli infermieri mostravano passione per la professione	Nurses showed passion for their profession.
Factor 4—Self-Directed Learning (Items 17–19)			
4	17	Mi sono stati offerti incontri sui miei bisogni di apprendimento	I was offered meetings focused on my learning needs.
4	18	Sono stato/a sollecitato/a ad elaborare il mio piano di autoapprendimento	I was encouraged to develop my self-directed learning plan.
4	19	Sono stato/a sollecitato/a ad auto-valutarmi	I was encouraged to self-evaluate.
Factor 5—Quality of the Learning Environment (Items 20–22)			
5	20	Questa sede è stata per me un buon ambiente di apprendimento	This clinical placement was a good learning environment for me.
5	21	Complessivamente sono soddisfatto/a della mia esperienza di tirocinio	Overall, I am satisfied with my clinical internship experience.
5	22	Vorrei tornare un giorno in questo contesto a lavorare	I would like to return to work in this setting one day.

Note: Response scale: 0 = Not at all (Per nulla); 1 = Somewhat (Abbastanza); 2 = Very much (Molto); 3 = Extremely (Moltissimo). Total score: sum of all 22 items (range 0–66). Factor scores: Factor 1 (items 1–6, range 0–18); Factor 2 (items 7–12, range 0–18); Factor 3 (items 13–16, range 0–12); Factor 4 (items 17–19, range 0–9); Factor 5 (items 20–22, range 0–9). English translations provided for international reference only; all data collection and scoring used the Italian version.
