# Supplementary material for: Resilience as a Predictor of Satisfaction and Well-Being in Nursing Clinical Education: A Cross-Sectional Study
Source: Nurs Rep. 2026 Apr 2;16(4):120. doi: 10.3390/nursrep16040120 (PMC13118439; doi:10.3390/nursrep16040120)

**Table S1.** STROBE Statement—checklist of items that should be included in reports of observational studies

(von Elm, E.; Altman, D.G.; Egger, M.; Pocock, S.J.; Gøtzsche, P.C.; Vandenbroucke, J.P. The STROBE statement: Guidelines for reporting observational studies. Int. J. Epidemiol. 2007, 36, 1495–1497. https://doi.org/10.1093/ije/dym158.)

| | **Item No** | **Recommendation** | **Page No** |
|---|---|---|---|
| **Title and abstract** | 1 | (*a*) Indicate the study's design with a commonly used term in the title or the abstract | 1 |
| | | (*b*) Provide in the abstract an informative and balanced summary of what was done and what was found | 1 |
| **Introduction** | | | |
| Background/rationale | 2 | Explain the scientific background and rationale for the investigation being reported | 2-3 |
| Objectives | 3 | State specific objectives, including any prespecified hypotheses | 3 |
| **Methods** | | | |
| Study design | 4 | Present key elements of study design early in the paper | 4 |
| Setting | 5 | Describe the setting, locations, and relevant dates, including periods of recruitment, exposure, follow-up, and data collection | 4 |
| Participants | 6 | (*a*) *Cross-sectional study*—Give the eligibility criteria, and the sources and methods of selection of participants | 4-5 |
| Variables | 7 | Clearly define all outcomes, exposures, predictors, potential confounders, and effect modifiers. Give diagnostic criteria, if applicable | 5 |
| Data sources/ measurement | 8* | For each variable of interest, give sources of data and dageils of methods of assessment (measurement). Describe comparability of assessment methods if there is more than one group | *5* |
| Bias | 9 | Describe any efforts to address potential sources of bias | 6 |
| Study size | 10 | Explain how the study size was arrived at | 6 + appendix A |
| Quantitative variables | 11 | Explain how quantitative variables were handled in the analyses. If applicable, describe which groupings were chosen and why | 6 |
| Statistical methods | 12 | (*a*) Describe all statistical methods, including those used to control for confounding | 6 |

| | | | |
|---|---|---|---|
| | | (*b*) Describe any methods used to examine subgroups and interactions | 6-7 |
| | | (*c*) Explain how missing data were addressed | n.a. |
| | | (*d*) *Cross-sectional study*—If applicable, describe analytical methods taking account of sampling strategy | 6 |
| | | (*e*) Describe any sensitivity analyses | n.a. |
| **Results** | | | |
| Participants | 13* | (a) Report numbers of individuals at each stage of study—eg numbers potentially eligible, examined for eligibility, confirmed eligible, included in the study, completing follow-up, and analysed | 7 |
| | | (b) Give reasons for non-participation at each stage | 5 |
| | | (c) Consider use of a flow diagram | n.a. |
| Descriptive data | 14* | (a) Give characteristics of study participants (eg demographic, clinical, social) and information on exposures and potential confounders | 7 + table 1 |
| | | (b) Indicate number of participants with missing data for each variable of interest | n.a |
| Outcome data | 15* | *Cross-sectional study*—Report numbers of outcome events or summary measures | *7-10* |
| Main results | 16 | (*a*) Give unadjusted estimates and, if applicable, confounder-adjusted estimates and their precision (eg, 95% confidence interval). Make clear which confounders were adjusted for and why they were included | 10 |
| | | (*b*) Report category boundaries when continuous variables were categorized | Tables |
| | | (*c*) If relevant, consider translating estimates of relative risk into absolute risk for a meaningful time period | n.a |
| Other analyses | 17 | Report other analyses done—eg analyses of subgroups and interactions, and sensitivity analyses | 10 |
| **Discussion** | | | |
| Key results | 18 | Summarise key results with reference to study objectives | 11 |
| Limitations | 19 | Discuss limitations of the study, taking into account sources of potential bias or imprecision. Discuss both direction and magnitude of any potential bias | 13 |
| Interpragetion | 20 | Give a cautious overall interpragetion of results considering objectives, limitations, multiplicity of analyses, results from similar studies, and other relevant evidence | 11-13 |
| Generalisability | 21 | Discuss the generalisability (external validity) of the study results | 13 |
| **Other information** | | | |

| Funding | 22 | Give the source of funding and the role of the funders for the present study and, if applicable, for the original study on which the present article is based | 14 |
|---|---|---|---|

.

**Figure S1: Resilience vs. Well-being**

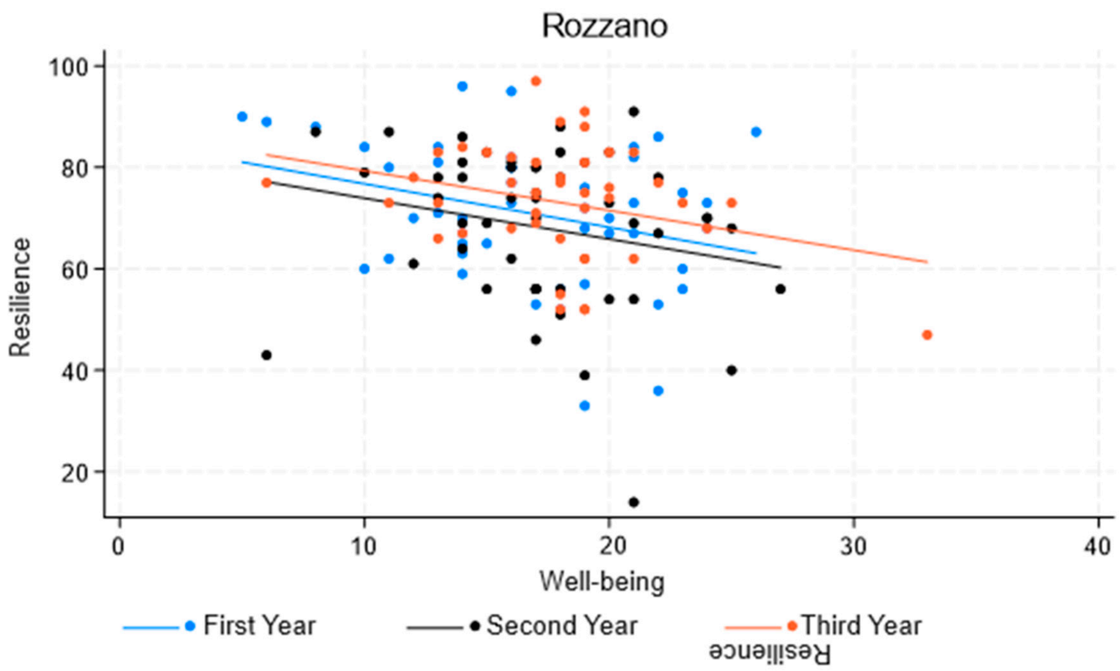


**Figure S2. Resilience vs. Well-being**

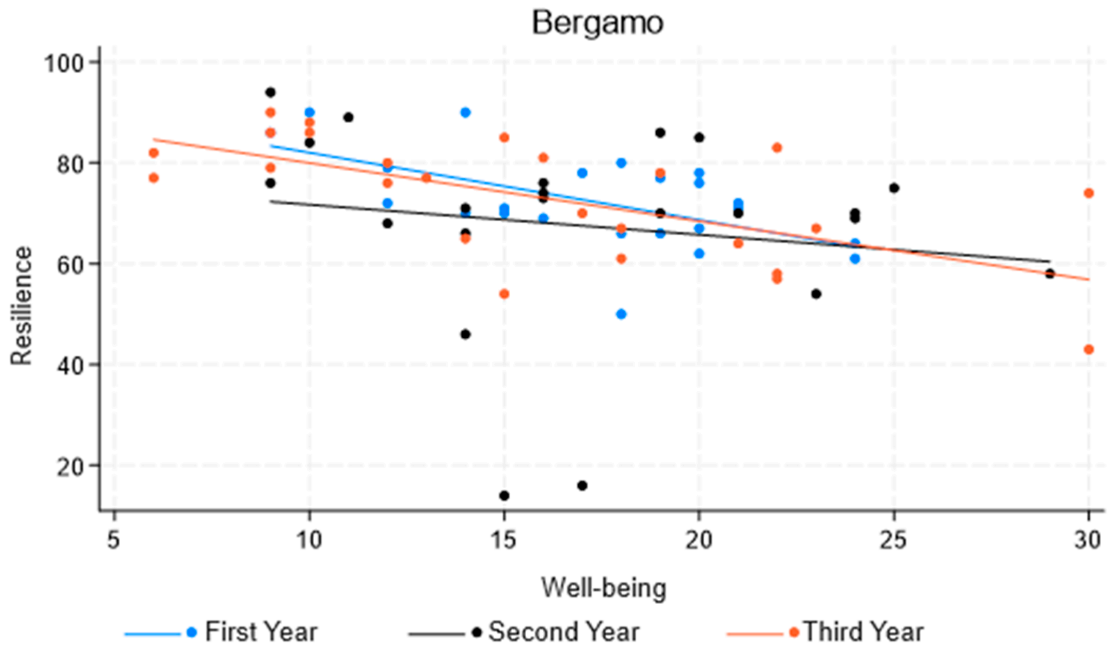

**Figure S3: Resilience vs. Well-being**

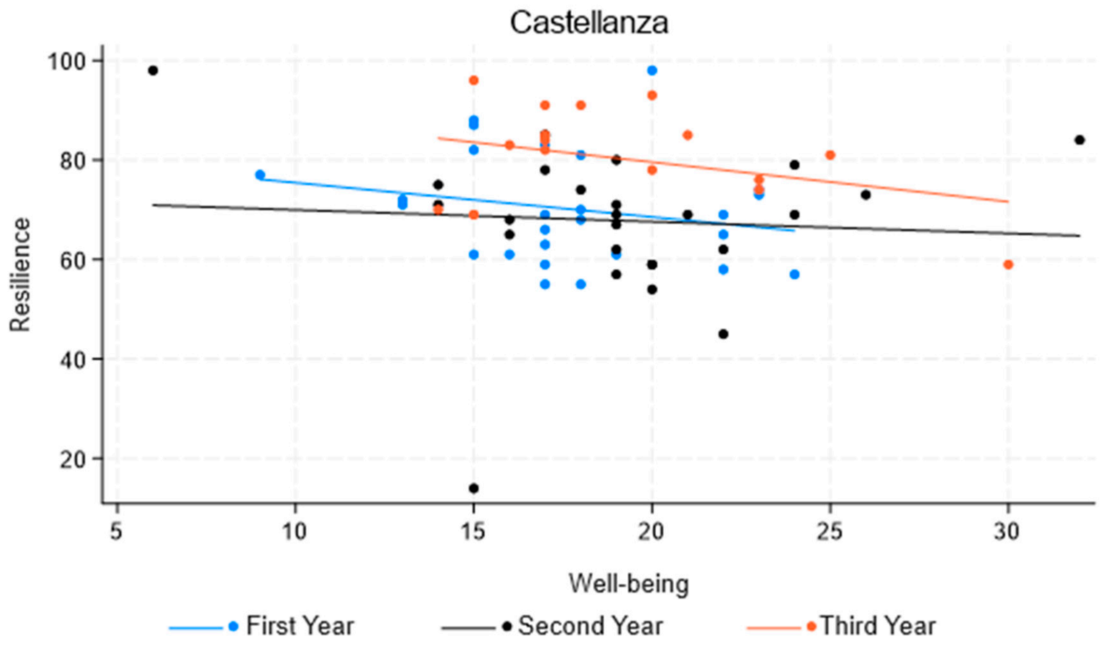


**Figure S4: Resilience vs. CLEQi**

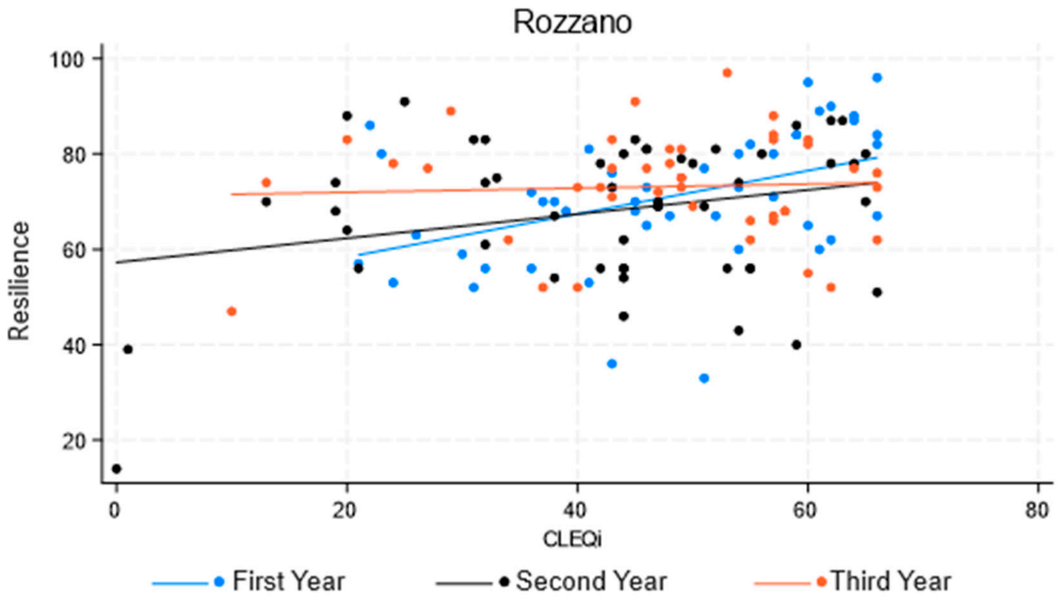

**Figure S5. Resilience vs. CLEQi**

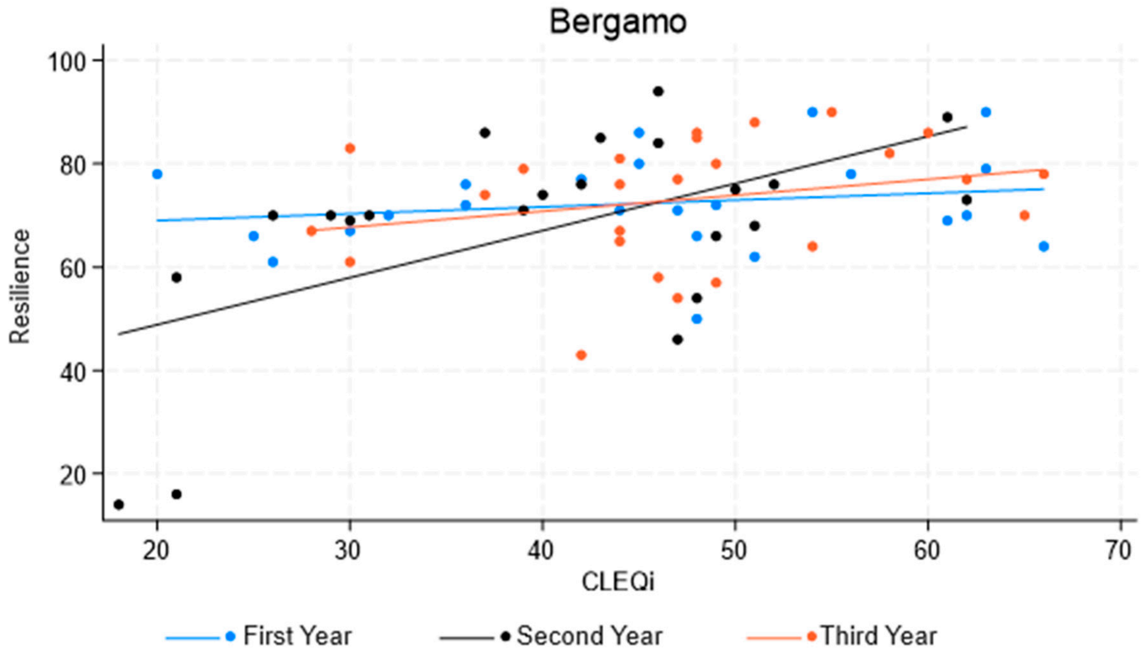


**Figure S6. Resilience vs. CLEQi**

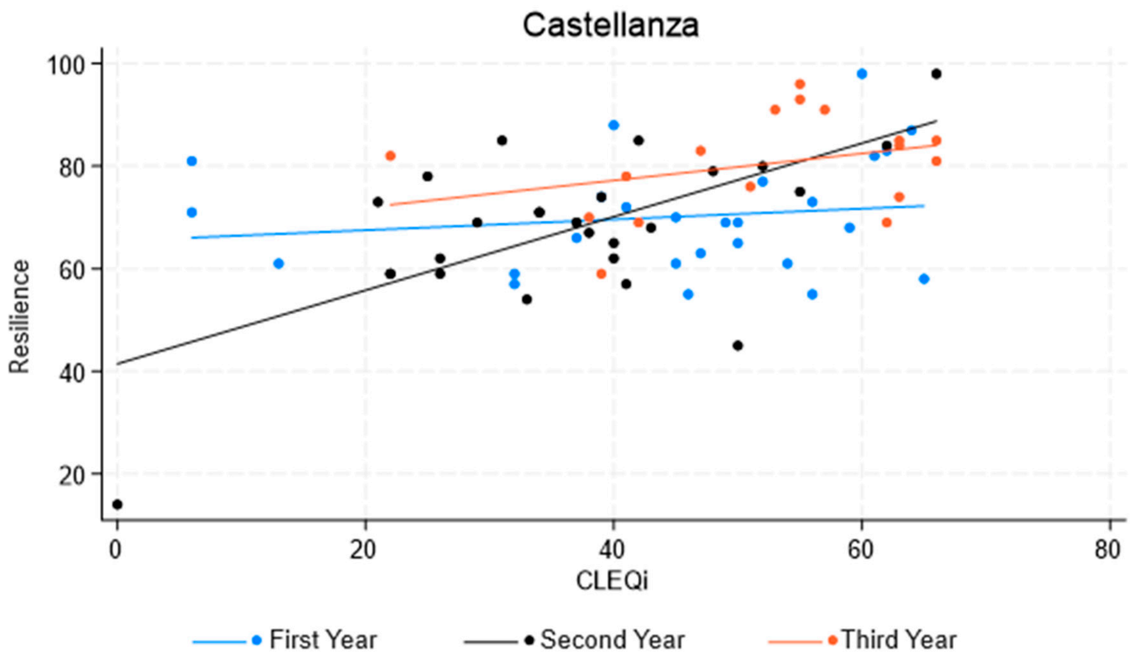

Supplement: Supplementary file 1 [file nursrep-16-00120-s001.zip › nursrep-4173237-supplementary.pdf]
